# Reducing Acneiform Rash Induced by EGFR Inhibitors With Honeysuckle Therapy: A Prospective, Randomized, Controlled Study

**DOI:** 10.3389/fphar.2022.835166

**Published:** 2022-02-18

**Authors:** Zhen Liu, Tian Tian, Binbin Wang, Demin Lu, Jian Ruan, Jianzhen Shan

**Affiliations:** ^1^ Department of Medical Oncology, the First Affiliated Hospital, Zhejiang University School of Medicine, Hangzhou, China; ^2^ Department of Medical Oncology, Zhejiang Hospital of Traditional Chinese Medicine, Hangzhou, China; ^3^ Department of Medical Oncology, the Second Affiliated Hospital, Zhejiang University School of Medicine, Hangzhou, China

**Keywords:** honeysuckle, acneiform rash, EGFR inhibitors, colorectal cancer, NSCLC

## Abstract

**Background:** Epidermal growth factor receptor inhibitors (EGFRIs), including cetuximab, erlotinib, gefitinib and icotinib, have been proven to be effective in treating colorectal cancer or lung cancer. However, most of patients who receive EGFRIs treatment experience cutaneous toxicities, such as acneiform or papulopustular rashes, which affects quality of life and leads to discontinuation of cancer therapies. Honeysuckle is a traditional herb historically used to treat skin rash for thousands of years in Eastern Asia and showed proven safety in human.

**Methods:** To investigate whether honeysuckle therapy could control EGFRIs induced acneiform rashes, a total of 139 colorectal and lung cancer patients with EGFRIs treatments were recruited in a prospective study. Patients were randomized to 3 arms (Arm A: prophylactic treatment with honeysuckle before rash occurred; Arm B: symptomatic treatment with honeysuckle when rash occurred; Arm C: conventional treatment with minocycline and a topical solution when rash occurred). The incidences, severities and recovery time of acneiform rash were observed in each arm.

**Results:** Honeysuckle treatment reduced incidences of EGFRIs induced acneiform rash, which were 56.5, 68.1 and 71.7% in Arm A, B and C, respectively (*p* = 0.280). Severities of rash (CTCAE grade 2 and 3) were significantly lower in prophylactic honeysuckle treatment (Arm A) compared to conventional treatment (Arm C) (*p* = 0.027), which was 10–21%, respectively. Patients with honeysuckle treatment recovered more quickly from pruritus, the median time was 22, 36 and 58 days in Arm A, B and C, respectively (*p* = 0.016).

**Conclusion:** Honeysuckle was effective in reducing incidences and severities of EGFRIs induced acneiform rash, especially for prophylactic treatment.

## Introduction

Epidermal growth factor receptor (EGFR) targeted therapies, including the monoclonal antibodies (mAbs) cetuximab and panitumumab, and the EGFR tyrosine kinase inhibitors (TKIs) erlotinib, gefitinib and lapatinib have been proved to be effective in a range of tumors, such as colorectal cancer (CRC), non-small cell lung cancer (NSCLC) and breast cancer (Xu et al., 2017). Theses EGFR inhibitors (EGFRIs) showed generally low hematological, gastrointestinal side-effects and hair loss compared with cytotoxic agents. However, the cutaneous toxicities, presented by itching, redness, swelling or pain, are more common during EGFRIs treatment, which occurs in 65–90% of patients (Fabbrocini et al., 2015).

Both classes of EGFRIs may induce cutaneous adverse events. The skin toxicities affect both patient quality of life (QoL) and treatment compliance. The severe adverse effects can also predispose the skin to bacterial, fungal, or viral infections, which leads to a discontinuation of the antineoplastic therapy (Braden and Anadkat, 2016).

The most common cutaneous side effect is acneiform rash (follicular papulopustular), which eruptions on the face, scalp, chest, and upper back. It is a dose-dependent skin toxicity, and usually develops in the first 1–2 weeks, peaks at 3–4 weeks on therapy, but can often persist over several months (Fabbrocini et al., 2015). Although a large number of patients receiving EGFRIs experience acneiform, few controlled studies have been conducted to determine the best practices for its management.

Honeysuckle (*Lonicera japonica Thunb*), a classical herb that has been utilized in China, Korea, Japan and other East-Asian countries for treating skin rash and influenza for thousands of years (Shang et al., 2011; He et al., 2013). It is used in traditional medicine owing to its pharmacological properties including anti-oxidation (Leung et al., 2006), anti-inflammation (Kang et al., 2010; Chen et al., 2012), anti-cancer (Leung et al., 2008; Liao et al., 2013), as well as anti-bacteria and anti-virus (Zhou et al., 2015; Lee et al., 2017). The aqueous extract of honeysuckle (chrysanthemum tea) can relieve fever and flu-like symptoms (Shang et al., 2011). Therefore, we designed this prospective, randomized, controlled clinical trial, aimed to investigated whether honeysuckle could reduce the incidence and severity of acneiform induced by EGFRIs. The data presented herein would provide a novel, easily conducted and affordable approach to treat EGFRIs induced skin toxicities.

## Materials and Methods

### Study Design and Treatments

The clinical trial was designed as a prospective, open-label, randomized, multicenter study and performed synchronously in the First Affiliated Hospital of Zhejiang University, the Second Affiliated Hospital of Zhejiang University and Zhejiang Hospital of Traditional Chinese Medicine from June 2017 to December 2020. To evaluated the efficacy of honeysuckle on EGFRIs induced acneiform, patients were randomized to 3 arms: prophylactic treatment (Arm A), symptomatic treatment (Arm B) and conventional treatment (Arm C) in a 1:1:1 ratio.

Arm A: Prophylactic treatment. Once EGFRIs therapy started, patients received the prophylactic treatment with decocted honeysuckle (10 g honeysuckle in 200 ml soup) orally twice daily. In case grade ≥1 acneiform rash occurred, external application of decocted honeysuckle was given in addition to oral treatment. For external application, 50 g honeysuckle was decocted in 1,000 ml water on soft fire for 10 min. Three or four-layer gauze was soaked in decocted honeysuckle that was cooled to 38°C. The gauze was then gently squeezed and was spread on the skin where rashes were located for 15 min, three times daily, until rashes recovered.

Arm B: Symptomatic treatment. When grade ≥1 acneiform rash occurred, patients started to be treated with decocted honeysuckle both orally twice daily and externally three times daily as described in Arm A.

Arm C: Conventional treatment. Patients in this group received only conventional treatment, that is minocycline at 100 mg per day, combined with a topical solution containing 2% clindamycin and 1% hydrocortisone, twice daily when grade ≥1 acneiform rash occurred.

The scale used for evaluating acneiform rash was the National Cancer Institute Common Terminology Criteria for Adverse Events (NCI-CTCAE) grading scale, version 4.03.

### Patients

Adult patients (age ≥18 years) with pathological diagnosis of metastatic NSCLC harboring activating EGFR driver mutations (treated with erlotinib, gefitinib or icotinib) and patients of metastatic colorectal cancer (CRC) with wild-type RAS gene (treated with cetuximab) were enrolled. The Eastern Cooperative Oncology Group (ECOG) performance status had to be 0 to 3. Adequate organ functions and an estimated life expectancy of more than 12 weeks were required. Patients with short-term usage of EGFRIs (less than 3 months) and concurrent skin diseases were excluded for analyses.

### Outcome Measures

The primary outcome measure was the incidences of EGFRIs-related acneiform rash among 3 arms. The secondary outcome measures included the duration from onset of grade ≥2 acneiform rashes relieved to grade 1 or 0 and the safety of honeysuckle treatment.

Patients were evaluated every 2–3 weeks, and were followed up for at least 6 months after EGFRI therapies. Adverse effects (AEs) were graded according to the NCI-CTCAE scale.

### EGFRI Therapy

Patients with CRC were treated with cetuximab at an initial dose of 400 mg/m^2^, then 250 mg/m^2^ per week subsequently. Patients with NSCLC harboring activating EGFR driver mutations were treated with erlotinib 150 mg po QD, gefitinib 250 mg po QD or icotinib 125 mg po TID according to National Comprehensive Cancer Network (NCCN) or Chinese Society of Clinical Oncology (CSCO) guidelines.

If grade ≥3 acneiform rashes occurred, EGFRI therapies were discontinued until the rashes reduced to grade ≤1.

### Statistical Analysis

Continuous data were presented as medians and ranges, whose differences were analyzed by using one-way ANOVA. Discrete data were presented as frequency or percentage, whose differences were evaluated using Fisher’s exact test or Pearsons’ chi-squared test. Calculation was performed by SPSS (version 21.0, IBM Corp). *p <* 0.05 was considered statistically significant.

### Ethics

The study conformed to the principles of good clinical practice guidelines (GCP). All patients provided written informed consents. The study protocol was approved by the Ethics Committee of the Second Affiliated Hospital of Zhejiang University with protocol #2016-IIT-111.

## Results

### Patient Characteristics

In total, 182 patients met the inclusion criteria and were recruited. 43 patients were excluding due to short-term usage of EGFRIs (less than 3 months), 139 patients were finally analyzed, with 46 or 47 patients in each arm. Demographic characteristics such as age, gender and diseases were balanced among 3 arms (Table 1).

**TABLE 1:** Demographic characteristics and baseline clinical parameters of the participants.

	**Arm A**	**Arm B**	**Arm C**	***p* Value**
	**(n = 46)**	**(n = 47)**	**(n = 46)**	
Age, years	53.2 ± 21.1	49.5 ± 20.5	54.7 ± 19.9	0.641
Gender (male), %	58.7%	59.5%	56.5%	0.538
Disease, n				0.739
CRC	14	11	11	
NSCLC	35	34	34	
EGFRI, n				0.821
Cetuximab	14	11	11	
Erlotinib	11	10	10	
Gefitinib	12	10	10	
Icotinib	12	14	14	
ECOG PS, n				0.742
0	14	12	13	
1	20	24	23	
2	9	9	6	
3(NSCLC)	3	1	3	

### Incidence of Acneiform Rash

The overall incidences of acneiform rash were 56.5, 68.1 and 71.7% in Arm A, B and C, respectively (Table 2). No grade 4 or 5 AEs occurred. Prophylactic treatment (Arm A) had the lowest overall incidence of acneiform rash, although no significant statistically differences were observed among 3 groups (*p* = 0.280). Particularly, CTCAE grade 2 and 3 rashes were significantly lower in prophylactic honeysuckle treatment (Arm A) compared to conventional treatment (Arm C) (*p* = 0.027), which was 10–21%, respectively. The incidences of acneiform rashes among different EGFRIs varied (Table 3). As shown in Table 2, the incidence of grade 2 and 3 in Arm A was 21.7%, much lower than Arm B (38.3%) and Arm C (45.6%), indicating that prophylactic treatment of honeysuckle could reduce grade 2 and 3 rashes in all drug groups.

**TABLE 2:** Incidence of acneiform rashes in each group.

	**Arm A**	**Arm B**	**Arm C**	***p* Value**
	**(n = 46)**	**(n = 47)**	**(n = 46)**	
Overall incidence, n (%)	26 (56.5)	32 (68.1)	33 (71.7)	0.280
Grade, n (%)				0.151
0	21 (43.5)	14 (29.8)	13 (28.3)	
1	15 (32.6)	15 (36.2)	12 (26.1)	
2	8 (17.4)	16 (34.0)	14 (30.4)	
3	2 (4.3)	2 (4.3)	7 (15.2)	

**TABLE 3:** Incidences and gradings of acneiform rashes with different EGFRIs treatment.

	**Cetuximab**	**Erlotinib**	**Gefitinib**	**Icotinib**	***p* Value**
	**(n = 36)**	**(n = 31)**	**(n = 32)**	**(n = 40)**	
Overall incidence, n (%)	27 (75.0)	23 (74.2)	20 (62.5)	20 (50.0)	0.080
Grade, n (%)	17 (47.2)	16 (51.6)	24 (75.0)	34 (85.0)	0.003
0/1					
2	13 (36.1)	11 (35.5)	7 (21.9)	6 (15.0)	
3	6 (16.7)	4 (12.9)	1 (3.1)	0 (0.0)	

Pruritus is a frequent symptom caused by acneiform rash, which usually leads to quality of life deteriorated. Patients with prophylactic treatment of honeysuckle (Arm A) experienced delayed onset of pruritus, the median time were 18, 16, and 14 days after initiation of EGFRI therapies in Arm A, B and C, respectively (Table 4). Patients with honeysuckle treatment also recovered more quickly, which were 22, 36 and 58 days after onset of pruritus in Arm A, B and C, respectively (*p* = 0.016).

**TABLE 4:** Incidence and grading of acneiform rash with pruritus.

	**No. of cases**	**Onset, median (day)**	**From onset until resolution, median (day)**
Arm A (n = 46)	10	18	22^*^
Arm B (n = 47)	11	16	36
Arm C (n = 46)	13	14	58

* *p =* 0.016 (Arm A vs Arm C).

**FIGURE 1:**
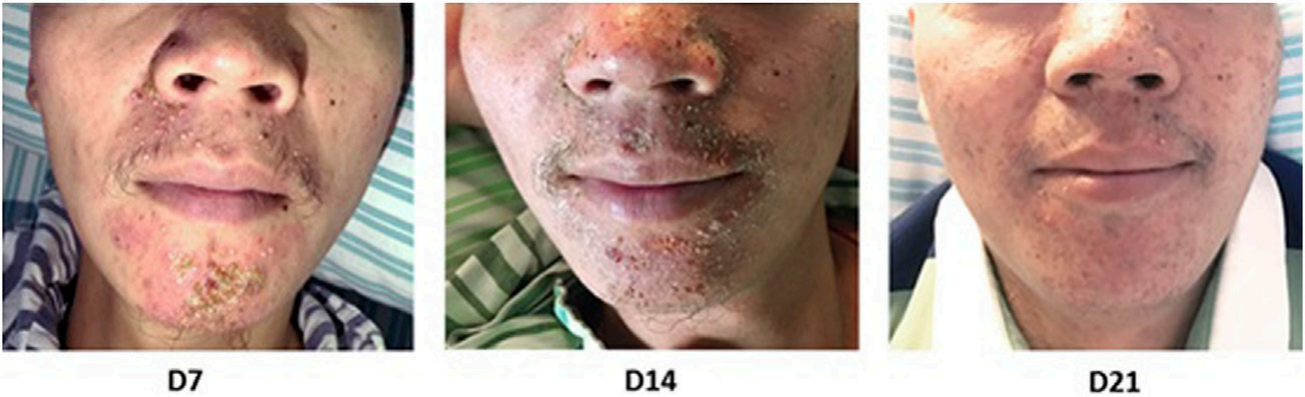
A representative patient in Arm B who recovered from grade 2 acneiform rash by honeysuckle treatment for 3 weeks.

Notably, 8 patients in Arm A, 5 patients in Arm B and 4 patients in Arm C experienced complete recovery of acneiform rashes (Figure 1).

### Safety

1 patient in Arm A and 2 patients in Arm B developed CTCAE grade 1 gastrointestinal discomfort after taking decocted honeysuckle orally. No other honeysuckle relevant AEs were found*.* Honeysuckle also did not increase EGFRIs related AEs.

## Discussion

EGFRIs have been proven to be effective in various types of solid tumors. In colorectal cancer, cetuximab, an IgG1 chimeric monoclonal antibody against EGFR, was associated with a significant improvement in overall survival (OS, hazard ratio [HR] for death, 0.77; 95% confidence interval [CI], 0.64 to 0.92; *p* = 0.005) and in progression-free survival (PFS, HR, 0.68; 95% CI, 0.57 to 0.80; *p* < 0.001) (Jonker et al., 2007). In NSCLC patients, EGFR tyrosine kinase inhibitors (TKIs) erlotinib, gefitinib and icotinib prolonged PFS significantly compared with chemotherapy (Pan et al., 2014; Batson et al., 2017). However, EGFRIs induced acneiform rash occurs in 65–90% of patients (Fabbrocini et al., 2015), and persistent pruritus is debilitating and severely affects quality of life, sometimes leads to a discontinuation of cancer therapy.

Honeysuckle is a classical herb that has been widely utilized with proven safety for treating skin rash and influenza for thousands of years in Eastern Asia. The aqueous extract of honeysuckle (chrysanthemum tea) is easily prepared. Besides low toxicity and extensive accessibility, the prices of herbal medicines are usually affordable. More importantly, the therapeutic effects of honeysuckle on acneiform rash were much better than conventional treatment, as we demonstrated in this study.

Acneiform rashes usually develop in the first 1–2 weeks after EGFRI therapy. In this study, we found that prophylactic treatment with honeysuckle showed promising efficacy in reducing the incidence and severity of EGFRIs induced acneiform rash. Therefore, prophylactic treatments are crucial, and appropriate medication should be considered throughout the whole course of EGFRI therapy aiming to minimize skin toxicities.

The mechanism of honeysuckle in reducing acneiform rashes is not clear. EGFR is expressed in the basal layer of the *epidermis*, which plays a crucial role in several cell activities, such as barrier function, inflammation and innate host defense (Lichtenberger et al., 2013). Obviously, EGFRIs inhibit both EGFR overexpressed in tumor cells and the one expressed in normal cells of the *epidermis*. EGFR inhibition induces the expression of chemokines that enhance skin inflammation through leukocyte recruitment, vascular dilation, and edema (Segaert and Van Cutsem, 2005; Lacouture, 2006). Feng *et al.* reported that cynaroside was the primary flavonoid component of honeysuckle, which alleviated serum levels of inflammatory factors including IL-1β and TNF-α, and suppressed the biomarker of pro-inflammatory macrophage M1 phenotype (iNOS+) and promotes the anti-inflammatory M2 polarization (CD206+) *in vivo* (Feng et al., 2021). Liu and his colleagues demonstrated that honeysuckle derived miR2911 down-regulated TGF-β1 promoted T lymphocytes infiltration (Liu et al., 2021). Totally, it is presumed that honeysuckle may exert the protective effects of acneiform rash mainly through its active ingredients of chlorogenic acid compounds, including chlorogenic acid and isochlorogenic acid.

However, due to the limited number of patients and short follow-up time, our understanding of honeysuckle is still limited. 139 patients were randomized to 4 EGFRIs treatment groups, 30–40 patients per each group. The analysis did not report influences of honeysuckle in terms of PFS and OS of patients. The detailed molecular mechanisms of honeysuckle also need further investigation.

## Conclusion

In summary, this prospective, randomized, controlled study suggested that honeysuckle is a promising treatment to reduce the incidences and severities of EGFRI-related acneiform rashes. Due to its low toxicity, extensive accessibility and affordable price, prophylactic treatment with honeysuckle is recommended for patients with EGFRIs therapies.

## Data Availability

The original contributions presented in the study are included in the article/supplementary material, further inquiries can be directed to the corresponding author.
